# A transformer-based multi-task deep learning model for simultaneous infiltrated brain area identification and segmentation of gliomas

**DOI:** 10.1186/s40644-023-00615-1

**Published:** 2023-10-27

**Authors:** Yin Li, Kaiyi Zheng, Shuang Li, Yongju Yi, Min Li, Yufan Ren, Congyue Guo, Liming Zhong, Wei Yang, Xinming Li, Lin Yao

**Affiliations:** 1 Department of Information, The Sixth Affiliated Hospital, Sun Yat-Sen University, Guangzhou, China; 2 School of Biomedical Engineering, Southern Medical University, Guangzhou, China; 3 Guangdong Provincial Key Laboratory of Medical Image Processing, Guangzhou, China; 4 Department of General Practice, The Sixth Affiliated Hospital, Sun Yat-Sen University, Guangzhou, China; 5 Department of Radiology, Zhujiang Hospital, Southern Medical University, Guangzhou, China

**Keywords:** Glioma, Deep learning, Tumor segmentation, Brain area Identification, Multi-task

## Abstract

**Background:**

The anatomical infiltrated brain area and the boundaries of gliomas have a significant impact on clinical decision making and available treatment options. Identifying glioma-infiltrated brain areas and delineating the tumor manually is a laborious and time-intensive process. Previous deep learning-based studies have mainly been focused on automatic tumor segmentation or predicting genetic/histological features. However, few studies have specifically addressed the identification of infiltrated brain areas. To bridge this gap, we aim to develop a model that can simultaneously identify infiltrated brain areas and perform accurate segmentation of gliomas.

**Methods:**

We have developed a transformer-based multi-task deep learning model that can perform two tasks simultaneously: identifying infiltrated brain areas segmentation of gliomas. The multi-task model leverages shaped location and boundary information to enhance the performance of both tasks. Our retrospective study involved 354 glioma patients (grades II-IV) with single or multiple brain area infiltrations, which were divided into training (*N* = 270), validation (*N* = 30), and independent test (*N* = 54) sets. We evaluated the predictive performance using the area under the receiver operating characteristic curve (AUC) and Dice scores.

**Results:**

Our multi-task model achieved impressive results in the independent test set, with an AUC of 94.95% (95% CI, 91.78–97.58), a sensitivity of 87.67%, a specificity of 87.31%, and accuracy of 87.41%. Specifically, for grade II-IV glioma, the model achieved AUCs of 95.25% (95% CI, 91.09–98.23, 84.38% sensitivity, 89.04% specificity, 87.62% accuracy), 98.26% (95% CI, 95.22–100, 93.75% sensitivity, 98.15% specificity, 97.14% accuracy), and 93.83% (95%CI, 86.57–99.12, 92.00% sensitivity, 85.71% specificity, 87.37% accuracy) respectively for the identification of infiltrated brain areas. Moreover, our model achieved a mean Dice score of 87.60% for the whole tumor segmentation.

**Conclusions:**

Experimental results show that our multi-task model achieved superior performance and outperformed the state-of-the-art methods. The impressive performance demonstrates the potential of our work as an innovative solution for identifying tumor-infiltrated brain areas and suggests that it can be a practical tool for supporting clinical decision making.

## Introduction

Gliomas are a type of malignant tumor that develops in the glial cells of the brain and spinal cord, and they are among the most lethal neurological malignancies [1, 2]. The World Health Organization (WHO) has classified glioma tumors into four grades according to their level of aggressiveness [3, 4]. Although advanced therapies have been developed for glioma treatment, neurological surgery remains the primary treatment modality to the survival rate of patients. The shape and size of gliomas can vary significantly based on their location in the brain and their growth rate. Besides, the definition of glioma boundaries is typically dependent on the on the expertise of the neuroradiologists. Manual segmentation and identification of glioma-infiltrated brain areas are extremely tedious and time-consuming. Therefore, there is a significant clinical need for automatic segmentation and identification of gliomas-infiltrated brain areas to aid in clinical decision-making, treatment planning, and ongoing tumor monitoring.

Magnetic resonance imaging (MRI) is a highly promising imaging technique due to its non-invasive nature. T2-fluid-attenuated inversion recovery (T2-FLAIR) abnormality is a reliable indicator of tumor progression, which has been found to correlate with impoved survival rate [5, 6]. Numerous innovative studies have proposed automatic segmentation the whole tumor using T2- FLAIR for volumetric measurement, radiomics, or radiogenomics [7, 8]. Furthermore, multi-task convolutional neural networks (CNNs) have been extensively proposed for tumor segmentation, while simultaneously addressing tasks such as IDH genotyping [9, 10], grading [11], molecular subtyping [11, 12], and detection of enhancing tumors [13]. For example, van der Voort et al. [11] developed a multi-task CNN (referred to as COM- Net for convenience) to predict the IDH mutation status, the 1p/19q co-deletion status, and the grade of a tumor, while simultaneously segmenting the tumor. Cheng et al. [10] proposed a transformer-based multi-task model (MTTU-Net) for glioma segmentation and IDH genotyping. Similar to our purposed method, the hybrid CNN-Transformer encoder is designed to extract shared local and global information for both glioma segmentation and IDH genotyping.

However, most studies have focused on glioma segmentation for clinical applications, with relatively little attention given to automatic identification of the precise anatomical location of glioma within the brain. It should be noted that the anatomical location of a glioma plays a crucial role in determining treatment options, clinical courses, and prognosis [14, 15].

**Fig. 1:**
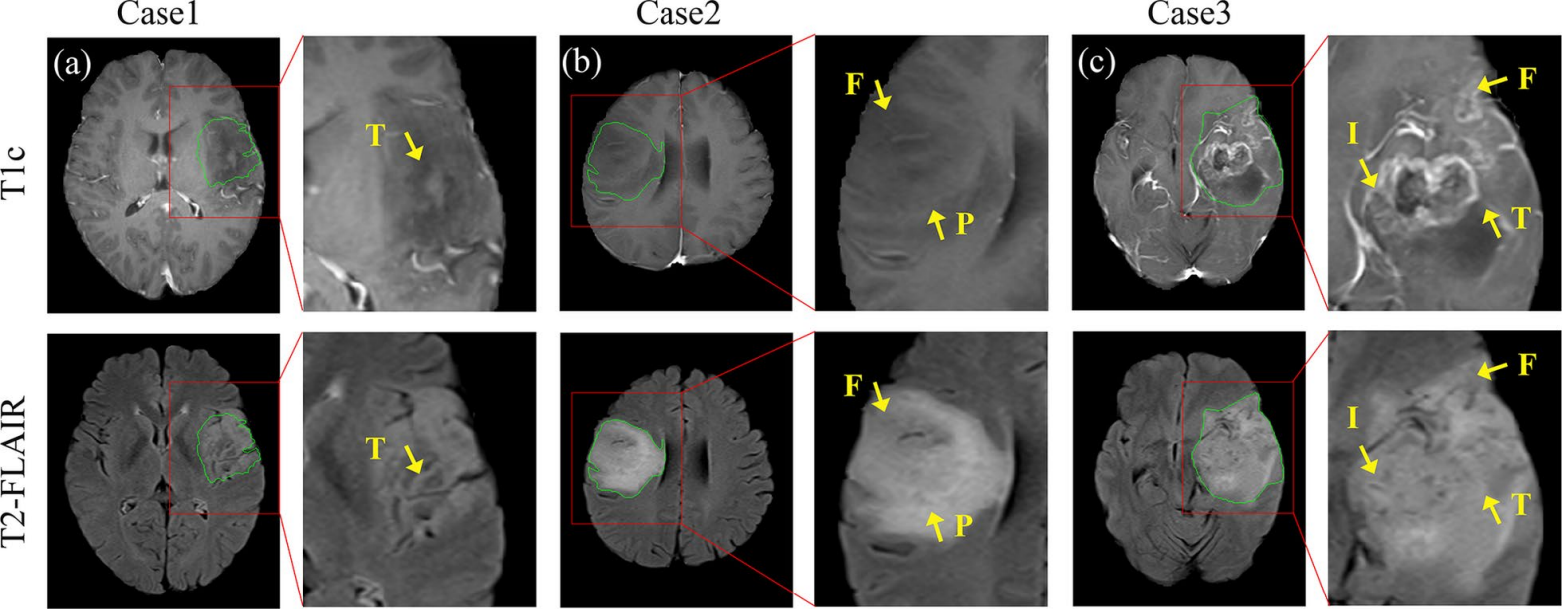
Examples of glioma patients with varying degrees of brain infiltration, including (**a**) infiltration of a single brain area; (**b**) infiltration of two brain areas, and (**c**) infiltration of three brain areas

A glioma tumor typically manifest in one or multiple brain areas (as shown in Figure (Fig. 1)), which can be consistently identified in the frontal lobe (F), parietal lobe (P), occipital lobe (O), temporal lobe (T), and insula lobe (I). Specifically, glioma in the insular area is particularly challenging in neurosurgical oncology [16]. Different tumor locations within these areas can have varying effects on tumor growth, symptoms, and treatment strategies, as previous studies have shown correlations between specific types and locations of tumors and clinical outcomes [15, 17]. The accurate identification of infiltrated brain areas holds important value in addressing challenges related to lesion anatomical localization, partial selection of surgical approaches (such as the use of the transcortical approach for frontal lobe lesions), and defining postoperative radiation therapy targets. However, identifying the glioma-infiltrated brain areas remains a challenging task due to the wide variation of tumor appearance. Unlike most multi-class classification tasks, identifying the glioma-infiltrated brain areas is a multi-label classification task, meaning that one subject can have one or more labels. For instance, as shown in Fig. 1, the glioma tumor can be found in a single brain area (Fig. 1(a)) or more than one brain areas (Fig. 1(b) and (c)). Although existing CNN models such as VGG16 [18], ResNet [19], EfficientNet [20], and Inception [21] have achieved significant performance in multi-class classification, accurately identifying the glioma-infiltrated brain areas without knowing the precise boundaries of the gliomas may be challenging for these models.

To address the aforementioned issues, we propose a transformer-based multi-task deep learning model that achieves simultaneous glioma segmentation and identification of infiltrated brain areas in an end-to-end framework. The main contribution of our study is the ability to accomplish these tasks simultaneously by sharing local and global features extracted from a hybrid CNN and Transformer network. Specifically, our multi- task deep learning framework leverages the boundary information from segmentation to enhance the performance of identifying glioma-infiltrated brain areas.

## Materials and methods

### Patients population

**Fig. 2:**
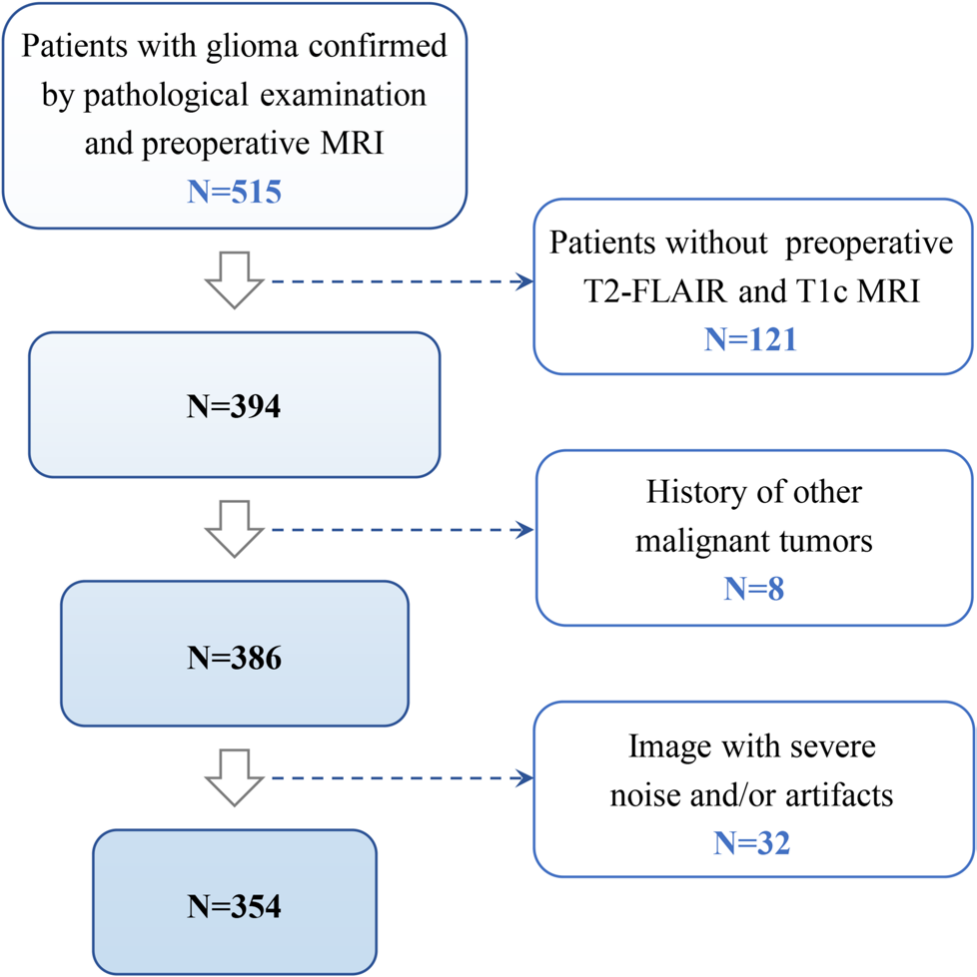
A Flowchart of patient selection

With the approval of institutional ethics committee, patients who received brain tumor resection and were diagnosed with glioma according to the 2016 WHO criteria [3] were retrospectively enrolled, following specific inclusion and exclusion criteria. The patient selection procedure in this study is shown in Fig. 2, and a total of 354 patients were included. The inclusion criteria included patients with glioma confirmed by pathological examination and preoperative MRI data. The exclusion criteria were as follows: (1) incomplete preoperative T2-FLAIR and post-contrast T1-weighted (T1c) MRI, (2) a history of other malignant tumors, and (3) images with severe noise and/or artifacts. Two MRI sequences, including T1c and T2-FLAIR, were acquired on three different types of scanners with two different field strengths, including Siemens 1.5 T, Siemens 3 T, Philips 1.5 T, Philips 3 T and GE 3 T. The in-plane pixel spacings of the T2FLAIR MR images range from 0.34 mm to 0.78 mm with an average of 0.60 mm and slice thicknesses range from 4.0 mm to 6.0 mm with an average of 5.05 mm. The in-plane pixel spacings of the enhanced T1 MR images range from 0.45 mm to 0.94 mm with an average of 0.65 mm and slice thicknesses range from 0.90 mm to 6 mm with an average of 4.85 mm. The images obtained by different scanners are randomly distributed in the training, validation and testing set.

The details of the demographic and clinical characteristics of patients, as well as the whole tumor cohort, are shown in Table 1.

**Table 1:** Demographic and clinical characteristics of patient cohort

	Training and validation sets		Independent test set	
	N	%	N	%
**No. of patients**	300		54	100
**Age**				
Range	16–82		11–71	
Average	47.38 ± 12.75		48.89 ± 14.28	
**Gender**				
Male	168	56	27	50
Female	132	44	27	50
**Grade range**	II-IV		II-IV	
II	149	49.67	21	37.04
III	59	19.67	14	22.22
IV	92	30.67	19	40.47
**Infiltrated brain areas**^**a**^				
frontal lobe (F)	172	57.33	29	53.7
parietal lobe (P)	65	21.67	11	20.37
occipital lobe (O)	36	12	4	7.41
temporal lobe (T)	115	38.33	27	50
insula lobe (I)	25	8.33	2	3.7
**Area counts**				
> = 1	300	100	54	100
> = 2	98	32.67	17	31.48
> = 3	15	5	2	3.7

^a^glioma-infiltrated brain areas, including frontal, parietal, occipital, temporal and insula lobe

### Data pre-processing

To reduce the effects of variability in acquisition and sequence parameters, image pre-processing was applied before analysis, which included MRI offset correction, image registration, skull stripping, and gray-level normalization. The N4ITK algorithm [22] was adopted to correct the low-frequency intensity non-uniformity of the magnetic field in the MR images. Rigid registration in FSL [23] and non-rigid registration in ANTs [24] were used to register the T1c and T2-FLAIR images for each patient. Image skull stripping was performed using BET in FSL. Gray-level normalization [25] was applied to adjust the gray values of MR images to compensate the MR scanner variability in intensity. The tumor masks were manually delineated around the tumor outline on 3D T2-FLAIR slices by radiologists with more than 10 years of experience with MRI. These tumor masks were used for training the tumor segmentation model. The glioma- infiltrated brain areas were labeled with 1 ~ 5 for the frontal lobe, parietal lobe, occipital lobe, temporal lobe, and insula lobe, respectively.

### Network details

**Fig. 3:**
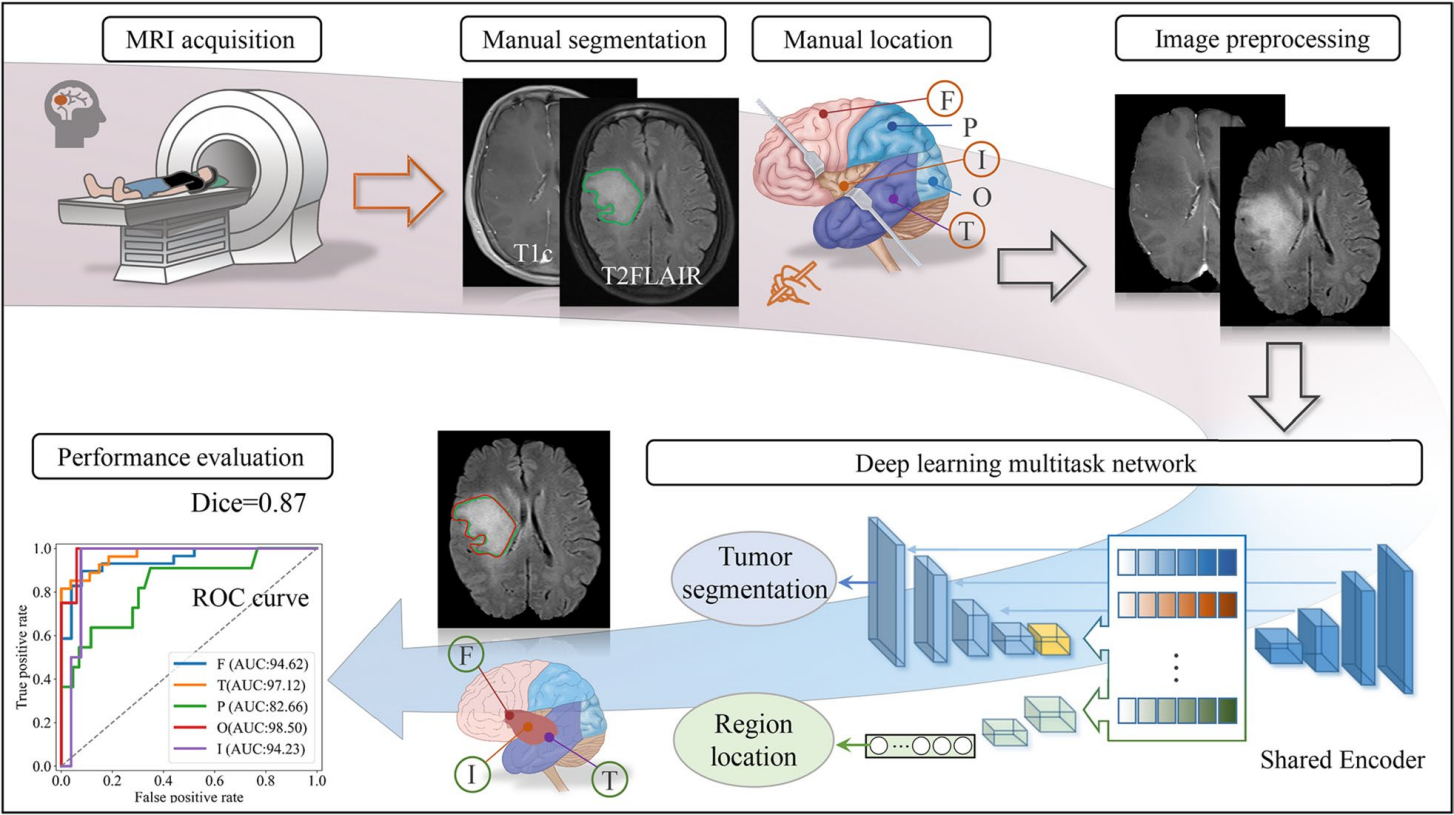
Overview of the proposed transformer-based multi-task deep learning model for infiltrated brain area identification and segmentation of gliomas

The architecture of our proposed transformer-based multi-task model for simultaneous infiltrated brain area identification and segmentation of gliomas is presented in Fig. 3. The network includes an encoder for learning the task relevant features, and two decoders for extracting task specific information. Besides, to capture the long- range dependencies and global information, a Swin Transformer module with convolutional layers in each head is embedded in the bottleneck of the encoder, which deeply fuses local and global features. The input, which concatenates T1c and T2-FLAIR images, is first fed into an initial convolution with a 3 × 3 × 3 kernel in the encoder. Then the task relevant features extracted from the encoder are separately processed by two decoders; one for performing voxel-level tumor segmentation and the other for identifying glioma-infiltrated brain areas using a classification head.

### Data partitioning and network implementation

The performance of the models was evaluated on an independent test set, which was not included in the model training. The remaining data were randomly divided into training and validation sets for parameter selection. After obtaining the optimal model parameters using the training and validation sets, the optimal model parameters were used to train the final model using the training data and validated on the independent test set.

The models were trained using PyTorch v1.7, with a data batch size of 4 and input image dimensions of 416 (*height*) × 352 (*width*) × 20 (*depth*). Gray-level normalization [25] was applied to adjust the gray values of MR images to compensate the MR scanner variability in intensity. Then they were linearly transformed to the range of [-1, 1]. Data augmentation techniques, including random rotation in the range of [-30, 30] and random scaling in the scale of [0.95, 1.05], were applied for model training. For optimization, we used the SGD optimizer with an initial learning rate of 0.0001 and trained the model for 200 epochs. To control the learning rate, we applied the step-decay learning rate control method, reducing the learning rate by 10 at the 100th and 160th epochs, respectively. We trained our models using cross-entropy loss for classification and a combination of cross-entropy loss and dice loss for segmentation. The weight ratios for segmentation were set to 0.6 and 0.4. To ensure a fair comparison, the compared models were trained with the same image size, learning rate, and number of iterations.

In addition, the two demographic characteristics (age and sex) and two clinical data variables (glioma grade and Karnofsky performance score) used in the comparison experiment were also pre-treated. Except sex was labeled with 0 and 1, the other three characteristics was standardized with z-score. Unlike image information, which is encoded using convolution, the binarized gender information is first transformed into one-dimensional features through Word Embedding. Prior to the fully connected layer, the three standardized clinical features (age, grade, and kps), the gender features represented in the embedding space, and the image features are concatenated together and collectively fed into the fully connected layer for the final prediction.

## Statistical analysis

All well-trained models were evaluated on both a validation set and an independent test set. The performance of multi-label classification model was assessed using various metrics, including the area under the receiver operating characteristic curve (AUC), accuracy (ACC), sensitivity (SEN), and specificity (SPE). Micro-averaging is used to describe all indexes of multi-label classification. The optimal threshold for the AUC value was determined by maximizing the sum of the sensitivity and specificity values. The 95% confidence intervals (CIs) were obtained using bootstrapping to assess variability. Besides, we used the Dice score (DSC), accuracy, sensitivity, specificity, Intersection over Union (IOU), Average Symmetric Surface Distance (ASSD), and 95th Percentile Hausdorff Distance (HD_95_) to evaluate the segmentation performance of the segmentation model. The DSC measures the degree of overlap between the ground truth and the predicted segmentation, with 100% indicate complete overlap.

## Results

### Patient characteristics

By applying specific inclusion and exclusion criteria, a total of 354 patients (195 males and 159 females) with an average age of 47.61 ± 12.99 years (range from 11 ~ 82 years) were included in our study. Of these, 270 were allocated to the training set, 30 to the validation set, and 54 to the independent test set. As shown in Table 1, 243 patients (68.64%) were diagnosed with low-grade gliomas (grade II and III), while 111 patients (31.36%) were diagnosed with high-grade gliomas (grade IV). Besides, a total of 239 gliomas (67.51%) were located within a single anatomic area, while 115 gliomas (32.49%) infiltrated across two or more areas. Specifically, the gliomas in the frontal lobe accounted for 56.78%, those in the parietal lobe accounted for 21.47%, those in the occipital lobe accounted for 11.30%, those in the temporal lobe accounted for 40.11%, and those in the insula lobe accounted for 7.62% of all patients.

### Model performance of glioma-infiltrated brain area identification

**Fig. 4:**
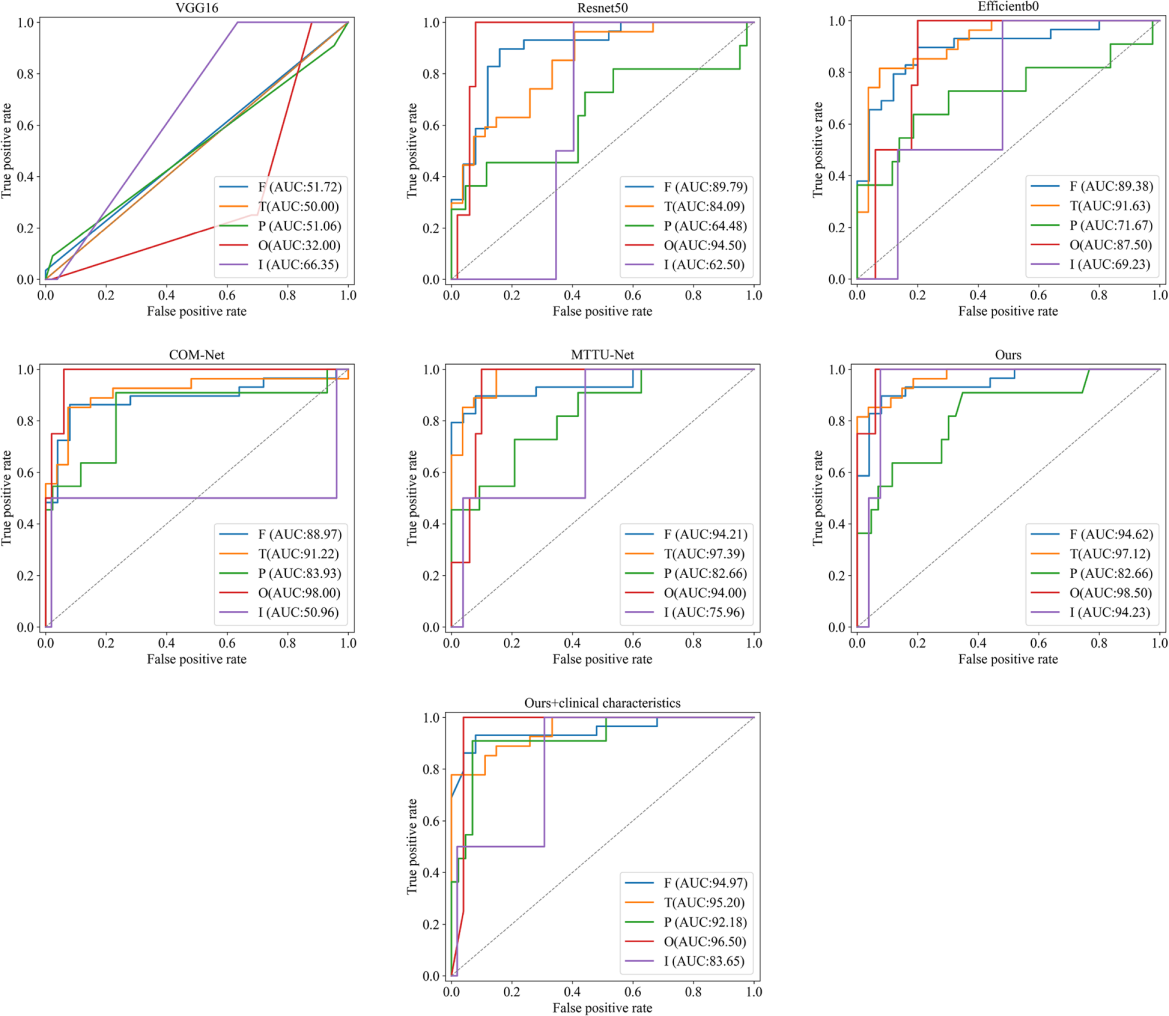
ROC curves of six distinct deep learning-based classification models, including VGG16, ResNet50, EfficientNetb0, COM-Net, MTTU-Net and the proposed method, for identifying the glioma-infiltrated brain areas. The seventh subplot evaluates our method enhanced with additional clinical characteristics

Tables 2 and 3 and Fig. 4 show the performance of identifying the glioma-infiltrated brain areas. Specially, Table 3 is adopted to show the performance of models for gliomas that infiltrated in single and multiple regions. Our proposed method achieves better performance than VGG16, ResNet50, EfficientNetb0 and COM-Net, MTTU-Net, with AUC of 94.95% (95% CI, 91.78–97.58) on the independent test set. Figure 4 illustrates the Receiver Operating Characteristic (ROC) curves for six distinct deep learning-based classification models, along with an enhanced iteration of our model that incorporates four additional clinical characteristics. These models were evaluated on an independent test set, yielding AUC values of 78.90% (95% CI, 73.07–84.28), 87.10% (95% CI, 81.73–92.04), 85.23% (95% CI, 78.75–90.74), 90.22% (95% CI, 85.12–94.61), 93.51% (95% CI, 90.22–96.37), 94.95% (95% CI, 91.78–97.58), and 95.07% (95% CI, 92.28–97.48) for VGG16, ResNet50, EfficientNetb0, COM-Net, MTTU-Net, our method, and our method with added clinical characteristics, respectively. Tables 2 and 3 also present the results of clinically related subgroups of patients. Our method outperforms the aforementioned state-of-the-art classification methods in terms of AUC, with an AUC of 95.25% (95% CI, 91.09–98.23) in grade II, an AUC of 98.26% (95% CI, 95.22–100.00) in grade III, an AUC of 93.83% (95% CI, 86.57–99.12) in grade IV, an AUC of 98.90% (95% CI, 97.46–99.94) in single infiltration (infiltrating brain area count), an AUC of 91.48% (95% CI, 84.27–97.08) in double infiltration and an AUC of 100% (95% CI, 100–100) in triple infiltration.

**Table 2:** Classification performance of the various models on both the validation and independent test set

		Validation set				Independent test set			
Data	Model	AUC (95% CI)	ACC	SEN	SPE	AUC (95% CI)	ACC	SEN	SPE
	VGG16	76.81(68.82–84.59)	72.41	71.43	72.82	78.90(73.07–84.28)	74.44	76.71	73.6
	Resnet50	89.20(82.50–95.03)	85.52	83.33	86.41	87.10(81.73–92.04)	83.33	76.71	85.79
	Efficientb0	88.58(82.34–93.55)	81.38	80.95	81.55	85.23(78.75–90.74)	79.63	76.71	80.71
All	COM-Net	88.65(81.84–93.77)	83.45	85.71	82.52	90.22(85.12–94.61)	85.93	82.19	87.31
	MTTU-Net	90.15(84.70–94.82)	85.52	78.57	88.35	93.51(90.22–96.37)	87.41	80.82	89.85
	Ours	91.42(86.19–95.63)	82.07	83.33	81.55	94.95(91.78–97.58)	87.41	87.67	87.31
	+ clinical characteristics	94.15(90.22–97.35)	88.28	88.10	88.35	95.07(92.28–97.48)	87.78	90.41	86.80
	VGG16	84.00(73.92–92.42)	80.00	84.00	78.33	78.10(68.76–87.02)	71.43	68.75	72.60
	Resnet50	91.33(84.54–97.06)	85.88	84.00	86.67	80.01(69.81–89.83)	75.24	68.75	78.08
	Efficientb0	91.80(84.90–97.13)	85.88	88.00	85.00	78.00(67.07–88.49)	73.33	78.12	71.23
Grade	COM-Net	91.73(85.28–97.20)	85.88	92.00	83.33	86.22(76.29–94.43)	80.95	75.00	83.56
II	MTTU-Net	91.13(84.36–96.74)	85.88	80.00	88.33	92.81(86.47–97.42)	84.76	78.12	87.67
	Ours	93.50(87.64–98.05)	85.88	80.00	88.33	95.25(91.09–98.23)	87.62	84.38	89.04
	+ clinical characteristics	98.07(95.39–99.82)	94.12	96.00	93.33	94.91(90.10–98.77)	91.43	90.62	91.78
	VGG16	63.89(40.26–85.58)	64.00	55.56	68.75	85.07(73.64–93.75)	80.00	93.75	75.93
	Resnet50	84.72(61.90–100)	88.00	88.89	87.50	94.21(88.00–98.72)	88.57	93.75	87.04
	Efficientb0	87.50(71.43–99.12)	84.00	77.78	87.50	94.56(87.21–99.23)	91.43	87.5	92.59
Grade	COM-Net	76.39(53.00–94.81)	76.00	77.78	75.00	97.34(93.06–100.00)	91.43	87.5	92.59
III	MTTU-Net	88.19(70.13–100)	84.00	77.78	87.50	96.18(90.06–100.00)	94.29	87.5	96.3
	Ours	90.28(73.81–100)	84.00	77.78	87.50	98.26(95.22–100.00)	97.14	93.75	98.15
	+ clinical characteristics	90.28(75.00–100)	84.00	66.67	93.75	97.22(92.45–100.00)	94.29	87.5	96.3
	VGG16	69.44(50.00–87.93)	60.00	50.00	62.96	76.43(66.57–85.84)	73.68	76.00	72.86
	Resnet50	89.35(76.67–98.67)	85.71	87.50	85.19	92.00(84.51–97.81)	86.32	84.00	87.14
	Efficientb0	82.41(63.33–97.06)	68.57	75.00	66.67	86.69(76.01–95.70)	81.05	80.00	81.43
Grade	COM-Net	89.35(76.53–98.67)	80.00	87.50	77.78	90.23(81.32–97.73)	89.47	88.00	90.00
IV	MTTU-Net	90.74(76.61–100)	82.86	87.50	81.48	92.86(86.76–97.63)	86.32	80.00	88.57
	Ours	91.44(80.10–98.98)	82.86	87.50	81.48	93.83(86.57–99.12)	87.37	92.00	85.71
	+ clinical characteristics	87.96(75.29–98.15)	88.57	87.50	88.89	93.94(88.17–98.19)	86.32	92.00	84.29

**Table 3:** Classification performance of the various models in single and multiple regions on both the validation and independent test set

		Validation set				Independent test set			
Number	Model	AUC (95% CI)	ACC	SEN	SPE	AUC (95% CI)	ACC	SEN	SPE
	VGG16	80.56 (68.09–90.84)	71.11	77.78	69.44	84.08 (78.08–89.24)	75.68	89.19	72.30
	Resnet50	88.73 (76.55–97.10)	87.78	88.89	87.50	93.22 (88.37–96.90)	89.19	89.19	89.19
	Efficientb0	90.74 (81.94–97.80)	84.44	83.33	84.72	91.36 (84.77–96.67)	84.86	86.49	84.46
One	COM-Net	92.21 (85.64–97.21)	83.33	88.89	81.94	97.55 (95.14–99.30)	91.89	91.89	91.89
	MTTU-Net	92.75 (86.13–97.84)	86.67	88.89	86.11	98.50 (96.49–99.85)	93.51	94.59	93.24
	Ours	94.02 (88.58–98.00)	86.67	88.89	86.11	98.90 (97.46–99.94)	96.76	94.59	97.30
	+ clinical characteristics	96.26 (91.93–99.47)	91.11	94.44	90.28	97.24 (94.83–99.21)	95.68	89.19	97.30
	VGG16	81.25 (66.35–93.81)	80.00	75.00	83.33	77.33 (66.20–86.97)	70.67	63.33	75.56
	Resnet50	91.15 (80.53–98.47)	85.00	87.50	83.33	81.26 (70.36–91.34)	76.00	73.33	77.78
	Efficientb0	91.41 (80.91–97.95)	85.00	87.50	83.33	78.89 (67.36–89.08)	73.33	76.67	71.11
Two	COM-Net	89.06 (76.69–97.99)	85.00	87.50	83.33	82.30 (70.99–91.90)	78.67	76.67	80.00
	MTTU-Net	92.45 (82.34–98.93)	80.00	81.25	79.17	87.56 (78.95–94.48)	78.67	73.33	82.22
	Ours	91.93 (81.45–98.96)	80.00	75.00	83.33	91.48 (84.27–97.08)	85.33	80.00	88.89
	+ clinical characteristics	95.18 (86.89–100)	92.50	87.50	95.83	94.44 (89.02–98.25)	89.33	86.67	91.11
	VGG16	58.33 (14.29–95.83)	60.00	50.00	75.00	66.67 (25.00–100)	80.00	66.67	100.00
	Resnet50	100 (100–100)	100	100	100	100 (100–100)	100	100	100
	Efficientb0	91.67 (66.67–100)	80.00	83.33	75.00	87.50 (55.56–100)	80.00	83.33	75.00
Three	COM-Net	70.83 (22.22–100)	70.00	66.67	75.00	79.17 (41.67–100)	70.00	66.67	75.00
	MTTU-Net	83.33 (47.62–100)	70.00	66.67	75.00	100 (100–100)	80.00	66.67	100
	Ours	95.83 (77.78–100)	80.00	66.67	100	100 (100–100)	100	100	100
	+ clinical characteristics	100 (100–100)	80.00	66.67	100	100 (100–100)	90.00	83.33	100

We also integrated clinical features into our model, specifically two demographic characteristics (age and sex) and two clinical data variables (glioma grade and Karnofsky performance score). The results presented in the table demonstrate an improvement of 2.73% in AUC, 6.21% in accuracy, 4.77% in sensitivity, and 6.8% in specificity compared to our original MR-only model for all grades on validation set.

### Model performance of tumor segmentation

**Fig. 5:**
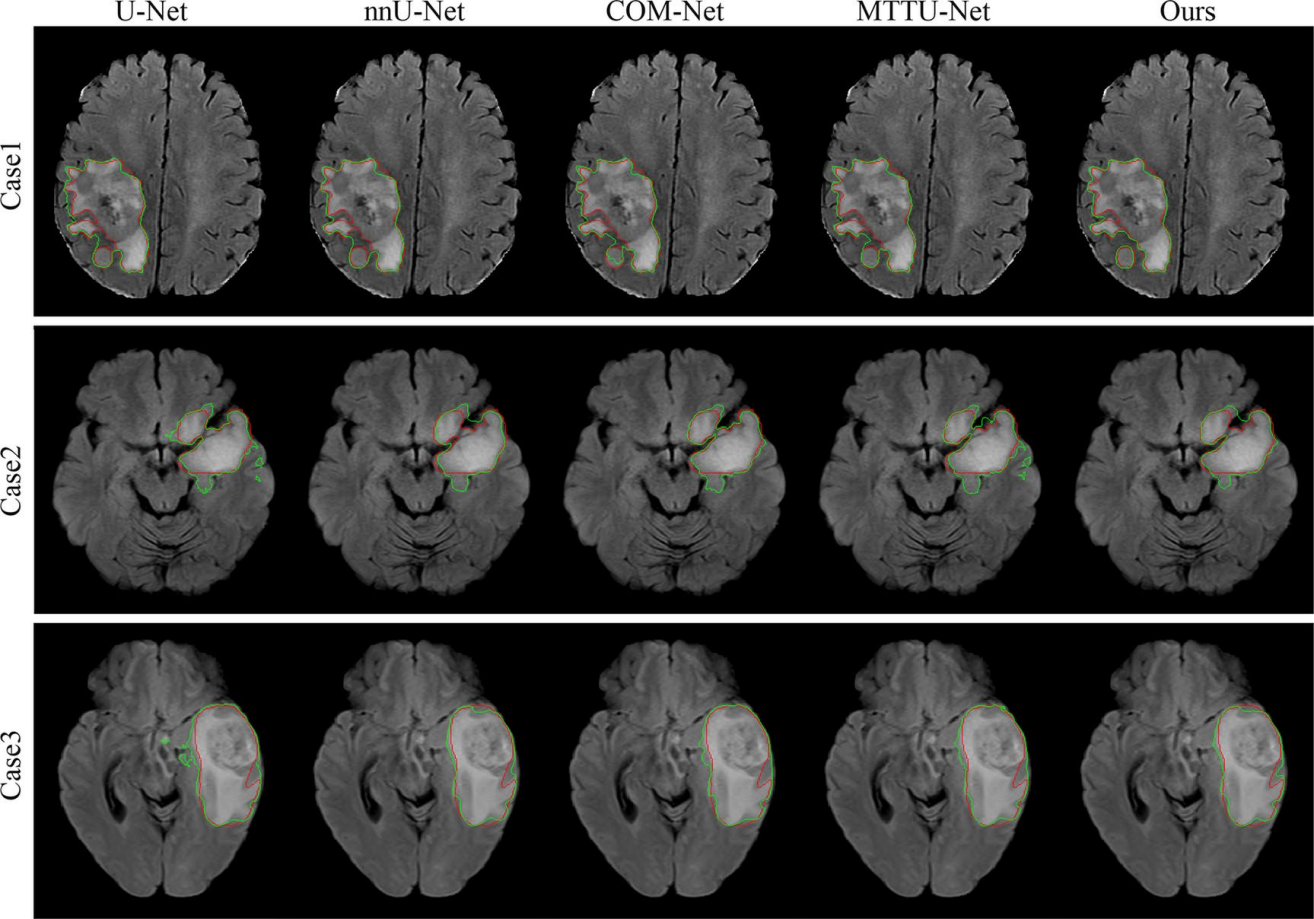
Visual segmentation results obtained from different segmentation methods. The red curve represents the ground truth, while the green curve shows the predicted segmentation

The performance of five different tumor segmentation methods, including U-net, nnUnet, COM-Net, MTTU- Net and our method was evaluated on the validation and independent test sets. The related results are presented Table 4 and Fig. 5, and the findings indicate that the proposed method outperforms the other four methods in terms of DSC for all grades of tumors on both the validation and independent test sets. Specifically, Fig. 5 shows that the proposed method achieved the highest overlap between the ground truth and the predicted segmentation, as indicated by the green curve. As shown in Fig. 5, our method achieves the highest overlap between the ground truth (red curve) and the predicted segmentation (green curve). Table 4 provides the numerical results of the evaluation, and it shows that the proposed method achieved the optimal DSC for all grades of tumors, including grade II, III, and IV. The DSC for the proposed method were 87.60% for all grades, which were higher than the corresponding scores for single-task methods (U-Net and nnUnet) and multi-task methods (COM-Net and MTTU-Net). Specifically, it achieved a DSC o 88.50% for grade II, 85.44% for grade III, and 88.20% for grade IV on the independent test set.

**Table 4:** Segmentation performance of the compared methods on both the validation and independent test set

		Validation set							Independent test set						
Data	Model	DSC	ACC	SEN	SPE	IOU	ASSD	HD_95_	DSC	ACC	SEN	SPE	IOU	ASSD	HD_95_
	U-Net	86.15	99.41	88.86	99.66	78.15	1.74	6.89	87.49	99.40	89.19	99.68	78.36	1.08	3.66
	nnUNet	86.49	99.42	86.95	99.73	78.63	1.15	4.81	87.58	99.40	87.10	99.77	78.37	0.59	2.95
	COM-Net	83.78	99.35	86.61	99.66	78.27	1.85	6.80	86.69	99.39	89.79	99.67	77.27	1.59	5.05
All	MTTU-Net	85.91	99.41	87.69	99.68	78.02	1.21	4.85	86.88	99.40	89.27	99.69	77.64	1.40	4.20
	Ours	86.22	99.41	87.93	99.69	78.86	0.65	2.55	87.60	99.40	89.10	99.71	78.40	0.70	2.63
	+ clinical characteristics	85.22	99.4	86.59	99.71	74.91	0.91	3.59	86.62	99.36	86.54	99.72	76.92	0.60	2.82
	U-Net	86.03	99.57	88.57	99.75	79.49	1.49	6.13	88.09	99.50	89.75	99.71	79.10	1.49	4.58
	nnUNet	87.48	99.63	87.79	99.83	80.54	0.49	2.19	88.43	99.54	87.55	99.80	79.53	0.58	2.29
	COM-Net	82.40	99.48	85.69	99.74	80.29	2.20	8.58	86.01	99.48	90.19	99.68	76.52	2.66	7.87
Grade	MTTU-Net	85.54	99.55	86.97	99.74	80.22	0.86	3.05	85.84	99.49	89.21	99.72	76.60	2.47	6.70
II	Ours	86.58	99.59	88.32	99.77	80.91	0.62	2.21	88.50	99.53	89.16	99.75	79.68	0.67	2.52
	+ clinical characteristics	85.12	99.57	87.2	99.78	74.88	1.06	4.12	87.70	99.51	87.52	99.74	78.52	0.62	3.01
	U-Net	85.99	99.22	87.94	99.60	78.35	1.36	3.15	85.09	99.42	87.82	99.68	75.26	1.01	4.23
	nnUNet	85.18	99.17	84.06	99.65	78.57	0.66	2.69	85.87	99.41	87.87	99.77	76.09	0.72	3.28
	COM-Net	84.09	99.12	86.08	99.55	78.63	1.62	5.66	85.38	99.38	90.84	99.64	75.42	1.30	4.84
Grade	MTTU-Net	86.02	99.20	87.62	99.64	78.73	0.70	3.00	86.16	99.41	89.32	99.69	76.36	0.88	3.12
III	Ours	86.64	99.25	88.65	99.60	78.89	0.69	2.61	85.44	99.37	90.19	99.66	75.4	0.90	3.45
	+ clinical characteristics	85.25	99.14	85.07	99.63	74.89	0.65	2.74	85.11	99.34	86.7	99.68	74.73	0.60	2.55
	U-Net	86.57	99.17	90.22	99.51	76.81	2.62	11.42	88.59	99.28	89.58	99.65	79.82	0.67	2.23
	nnUNet	85.04	99.09	86.99	99.55	76.94	3.09	12.68	87.90	99.24	86.04	99.75	78.75	0.51	3.43
	COM-Net	86.90	99.19	89.23	99.56	76.24	1.15	3.29	88.41	99.29	88.57	99.69	79.46	0.61	2.07
Grade	MTTU-Net	86.72	99.21	89.47	99.55	75.62	2.41	10.54	88.55	99.29	89.31	99.66	79.73	0.59	2.24
IV	Ours	85.02	99.10	86.47	99.58	76.98	0.71	3.36	88.20	99.28	88.22	99.70	79.20	0.59	2.15
	+ clinical characteristics	85.43	99.15	86.21	99.59	74.99	0.74	2.91	86.55	99.20	85.34	99.72	76.77	0.57	2.81

### Model performance of glioma-infiltrated brain area identification vs. experts

**Fig. 6:**
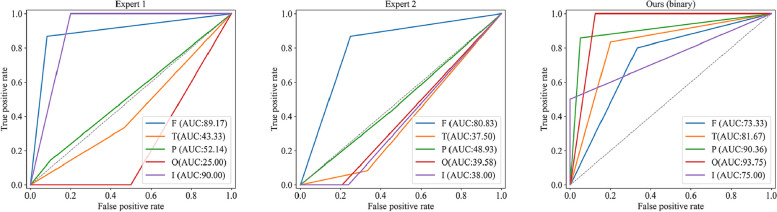
ROC curves of two experts and proposed model

To compare the accuracy of infiltrating tumor lobe classification by human and the model, we conducted a comparison experiment with a random sample of 50% in independent test set. Two experts dedicated to annotate glioma-infiltrated brain lobes based on enhanced T1 and T2-FLAIR MRI data (X.M.L and M.L with 10 years and 4 years of experience, respectively). They consecutively and independently evaluated the MR data from independent test set. The results of our model have been binarized for fair comparison. Table 5 shows performance of identifying glioma-infiltrated brain areas with AUCs of 61.58% (95% CI, 52.38–70.33), 57.21% (95% CI, 48.10–66.49) and 85.30% (95% CI, 77.71–91.70) for two experts and our model. The time spent in each case was 50 s and 2 min, respectively, which was much higher than the 0.4 s of the model. As shown in Fig. 6, the experts' results show largely individual differences. The two experts had a high sensitivity to the frontal lobe, while they could not discriminate well in the parietal, occipital, and temporal lobes. In contrast, although our model cannot achieve optimal performance in every category, it was able to achieve a higher level of discrimination in each brain lobe, with a better overall ability.

**Table 5:** Model performance of glioma-infiltrated brain area identification vs. experts

	Expert 1	Expert 2	Our Model
Time	50 s	2 min	0.4 s
AUC	61.58%	57.21%	85.30%
(95% CI)	(52.38–70.33)	(48.10–66.49)	(77.71–91.70)
ACC	65.93	62.96	86.67
SEN	51.28	43.59	82.05
SPE	71.88	70.83	88.54

### Visualization of local and global information of our model

**Fig. 7:**
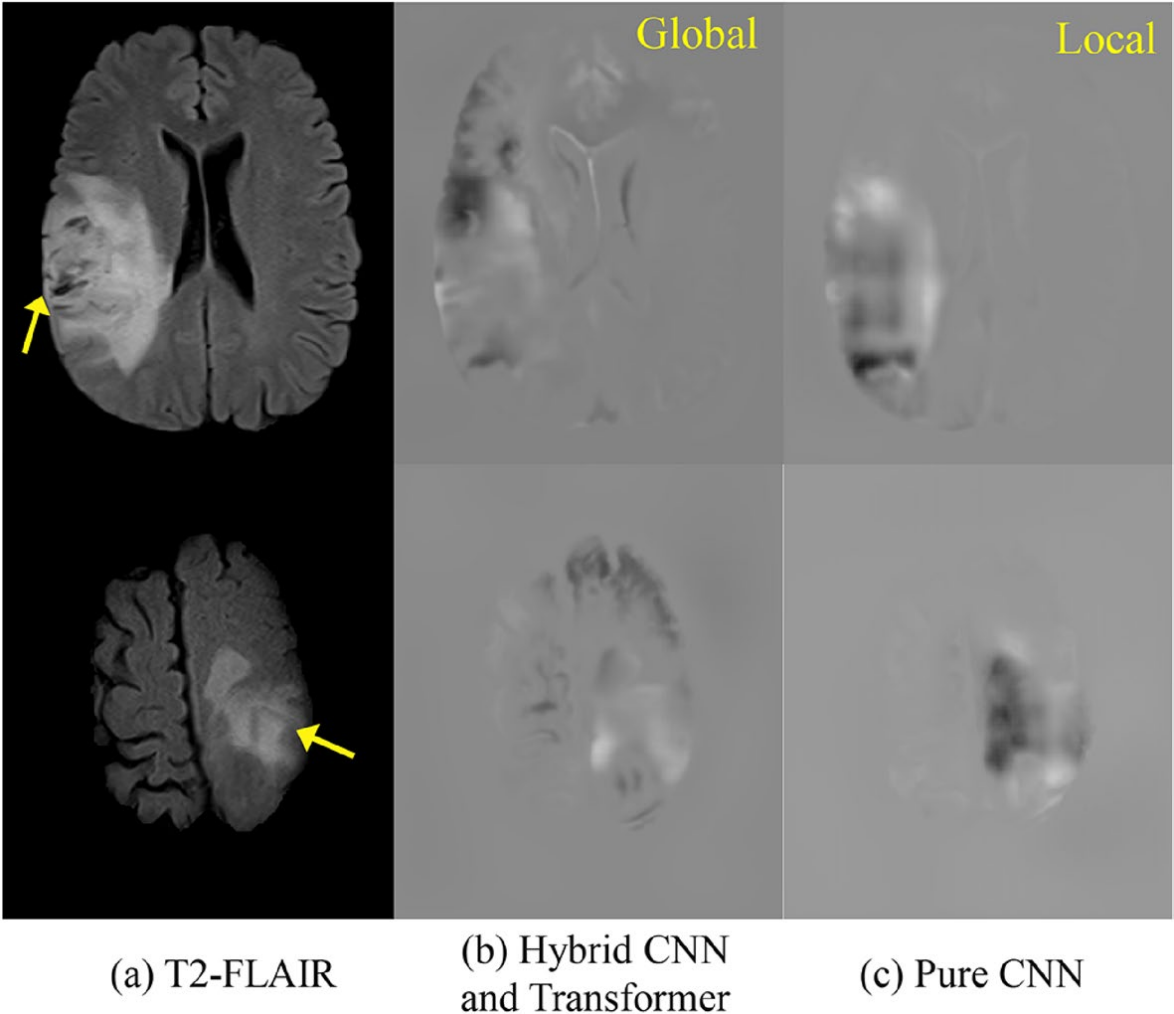
Guided backprop maps of (**b**) hybrid CNN and Transformer network and (**c**) pure CNN models. Illustrate the model's attention to both global and local information

To validate the extraction of specific global and local features, we compare the performance of the hybrid CNN and Transformer network with a pure CNN network using guided backpropagation [26] for visual interpretation. The results of this comparison were shown in Fig. 7. Given an input image, we perform the forward pass to the last convolutional layer we are interested in, then set to zero all activation except one and propagate back to the image to get a reconstruction. As shown in Fig. 7, the Guided Backpropagation map (Guided Backprop) reflects the pixels where the network is focused, with white indicating high attention and black low attention.

The pure CNN network shows attention to local tumor information, as evidenced by the high intensity in the tumor region (Fig. 7(c)). In contrast, the hybrid model, as shown in the Fig. 7(b), exhibits high attention regions associated with tumors and brain edges. This indicates that the hybrid model effectively learns global features, specifically the relative position relationship between tumors and brain edges. The corresponding class activation maps of the hybrid model provide insights into the decision regions when predicting infiltrating brain regions. By combining information from both global and local features, the hybrid model maximizes the advantages of each. The global features provide important information about the relative positions of tumors and brain edges, while the local features focus on specific tumor regions. This combination allows the hybrid model to achieve more accurate predictions of infiltrating brain regions.

## Discussion

In this study, we have developed a method that can predict the glioma-infiltrated brain areas while simultaneously achieving the tumor segmentation. Our approach incorporated the CNN and transformer network to leverage both local and global features, thus enhancing the performance of classification and segmentation.

Our method can automatically identify the tumor-infiltrated brain areas and provide a whole tumor segmentation, which can aid in determining the appropriate surgical approach and predict the prognosis [14, 15]. When the tumor infiltrates critical brain areas, the prognosis may worsen due to the increased risk of neurological deficits and complications. Our method provides an alternative for marking the specific tumor location. Besides, the predicted whole tumor segmentation can facilitate tumor volumetric measurement [5, 27], radiomics [28], or radiogenomics [29]. With short running time and reliable information about the areas of glioma infiltration and segmentation, our model can reduce the burden of clinicians and improve the efficiency of making more informed decisions about treatment strategies. In addition, the model's ability to perform these tasks based solely on MR images streamlines workflow and reduces reliance on other procedures. This makes the model readily applicable to a variety of clinical settings where MRI is routinely performed. The proposed model has the potential to improve patient outcomes, optimize treatment strategies, and contribute to the overall management of glioma patients.

The precise characterization of glioma is critical for clinical treatment plans [11]. Previous studies [5, 7, 8, 10, 11, 27] have developed deep learning methods for predicting the genetic or histological features of glioma, or for automatically delineating the tumor. Additionally, providing accurate information about the location of tumor-infiltrated brain areas is crucial for clinical decision making. Studies have shown the incidence of gliomas is related to anatomic locations [14, 30]. To the best of our knowledge, few studies have focused on achieving the identification of tumor-infiltrated brain areas and segmentation of the tumor at the same time. We develop a multi-task network to learn the relevant information between different tasks, enabling us to achieve both goals.

In contrast to molecular subtyping or grading of glioma, where each patient has a single label for a task, our infiltrated brain area identification task may have one or more labels per patient. Directly adapting the commonly used deep learning-based multi-class classification models, such as ResNet50 and VGG16, to the multi-label task may not be effective due to the irregular shape of gliomas. While adding multi-sequences of MRI images can help improve the performance of infiltrated brain area identification, the improvement is limited without the prior information about the glioma boundary. There are several factors that may explain our proposed method achieved higher performance of identifying glioma- infiltrated brain areas compared with other methods. First, unlike the aforementioned methods that perform classification and segmentation separately, our method uses a single encoder to extract the task-relevant features. Learning shared representations across different tasks can help prevent overfitting on each task and improve overall performance on all tasks. Second, our method co-optimizes the loss functions of brain area identification and tumor segmentation, improving the model's generalization ability and potentially solving label imbalance problems via shared label information. Third, transformer blocks were embedded into CNN-based network to address the intrinsic locality limitation of convolution operations.

Despite the superior performance of the validation and independent test sets, our study has several limitations. The main drawback is its retrospective nature, which makes it vulnerable to selection and recall bias, thereby potentially impacting the reliability and generalizability of the study findings. Our future direction encompasses the implementation of prospective studies, enabling the longitudinal follow-up of patients from the onset and facilitating the collection of real-time data for precise and comprehensive analysis. This approach aims to enhance the validity and robustness of our model while augmenting its applicability in clinical settings. Besides, advanced MRI sequences, such as diffusion or perfusion weighted imaging MRI, were not included in this study. While these sequences are not the clinical imaging routine for structural scans. Our results in Tables 2 and 3 indicate that superior performance can be achieved by using the T2-FLARI and T1c MRI. This method makes it easier to collect and obtain data and makes the model simpler but it may still limit the performance of the model. Given the increasing use of these advanced MRI sequences in clinical practice, we believe that integrating them into our future research will further improve the model's accuracy. It is essential to note that our study is limited to brain area identification and tumor segmentation by developing a single model. However, we acknowledge the potential of our model in other glioma-related prediction. In our future work, we aim to integrate the prediction of 1p/19q co-deletions, IDH mutations, and molecular subtyping to provide a more comprehensive analysis. By integrating these additional factors, we can deepen our understanding of gliomas at the molecular level and contribute to personalized treatment strategies and rognostications, enhancing the overall management of patients with gliomas.

In addition, we acknowledge the significance of detailed structural labeling, including deeper brain structures like the internal capsule. Future research will focus on enhancing the capabilities of our model to enable more accurate and detailed labeling of brain structure and function. We will incorporate advanced techniques and expand the scope of our model to encompass a broader range of anatomical and functional regions.

## Conclusion

In conclusion, we have developed a transformer-based multi-task deep learning model that can perform two critical tasks: identifying tumor-infiltrated areas of the brain and segmenting the tumor based on preoperative MR scans. Our model has demonstrated superior performance compared to state-of-the-art classification and segmentation models on both validation and independent test sets. It is believed that our model holds significant potential as a practical tool to assist clinicians in accurately identifying glioma-infiltrated areas and supporting treatment decisions.

## Data Availability

None.

## Abbreviations

AUC: Area under the ROC curve
CI: Confidence intervals
ACC: Accuracy
SEN: Sensitivity
SPE: Specificity
WHO: World Health Organization
MRI: Magnetic resonance imaging
T2-FLAIR: T2-fluid-attenuated inversion recovery
T1c: Post-contrast T1-weighted MR
CNN: Convolutional neural network
